# The effect of physical activity on depression in university students: the mediating role of self-esteem and positive psychological capital

**DOI:** 10.3389/fpsyg.2024.1485641

**Published:** 2024-09-24

**Authors:** Xiaolong Wei, Zhijie Lai, Zhaowen Tan, Ziyue Ou, Xueyou Feng, Guoqin Xu, Dongsheng Ai

**Affiliations:** ^1^School of Physical Education and Sport Science, Fujian Normal University, Fuzhou, China; ^2^School of Physical Education, Guangzhou College of Commerce, Guangzhou, China; ^3^School of Physical Education, Nanjing Xiaozhuang University, Nanjing, China; ^4^Department of Sports and Health, Guangzhou Sport University, Guangzhou, China; ^5^School of Clinical Medicine, Hainan Vocational University of Science and Technology, Haikou, China

**Keywords:** physical exercise, depression, self-esteem, positive psychological capital, college students

## Abstract

**Objective:**

This study aimed to investigate the relationship between physical exercise and depression among college students, focusing on the mediating role of self-esteem and positive psychological capital.

**Methods:**

Group psychological measurements were conducted on 579 students using various scales, including the Physical Activity Rating Scale (PARS-3), Self-Esteem Scale (SES), Positive Psychological Capital Questionnaire (PPQ), and Self-rating Depression Scale (SDS). The data was analyzed using SPSS 26.0 and bootstrap methods to test and analyze the effects.

**Results:**

A negative correlation between physical exercise and depressive mood, with physical exercise significantly predicting college students' depressive mood. Additionally, physical exercise was found to positively predict self-esteem and positive psychological capital, both of which are negatively predictive of depressive mood. Self-esteem and positive psychological capital were identified as significant mediators between physical exercise and depressive mood, with three mediating paths: physical exercise, self-esteem, and depressed mood (Path 1), exhibited an indirect effect of 0.017, with the bootstrap 95% confidence interval excluding 0 (LLCI = −0.051, ULCI = −0.004) and accounting for 8.30% of the total effect. Physical exercise, positive psychological capital, and depression emotion (Path 2), had an indirect effect of 0.049, with the bootstrap 95% confidence interval also not containing 0 (LLCI = −0.088, ULCI = −0.011) and contributing to 23.90% of the total effect. Physical exercise, self-esteem, positive psychological capital, depressed mood (Path 3), demonstrated an indirect effect of 0.006, with the bootstrap 95% confidence interval excluding 0 (LLCI = −0.011, ULCI = −0.001) and representing 2.90% of the total effect.

**Conclusion:**

Physical exercise negatively predicts depressive mood among college students and has a mediating effect through self-esteem and positive psychological capital, creating a chain-like impact on their depressive symptoms.

## 1 Introduction

The fast-paced nature of modern society has led to significant lifestyle changes, which offer both convenience and the potential for psychological issues, including depression. Depression is a complex negative emotion characterized by fatigue, low energy, feelings of worthlessness, and, in severe cases, suicidal thoughts. These issues contribute to increased pressure and socioeconomic burdens on various demographic groups (Wegner et al., 2020). College students, characterized by their high energy and active thinking, are viewed as the future leaders of both the economy and society. However, they frequently lack robust psychological coping mechanisms and social experience. When faced with challenges in academics, careers, and relationships during these formative years, their inability to effectively manage emotions can result in disappointment and depression. Research indicates an average prevalence of 20.8% of depression within this demographic from 2013 to 2023, underscoring the significant incidence of this mental health issue among college students (Gao et al., 2020; Chen et al., 2022). Prolonged periods of depression and disappointment can escalate from subclinical to pathological depression, adversely affecting adolescents' academic performance and diminishing their interest in activities, potentially leading to suicidal thoughts and behavior (Zhang et al., 2023; Grant et al., 2023). Consequently, identifying effective interventions to reduce depression rates among college students has become a critical focus for the Chinese government and various academic disciplines.

Physical activity is a prominent non-pharmacological intervention for preventing and treating depression (Khan and Brown, 2015). Engaging in appropriate physical activity fosters positive psychology, reduces anxiety and stress, and has a strong antidepressant effect (Tsai et al., 2003; Tang et al., 2009; Xu and Li, 2017). Although the antidepressant effect of physical exercise has been widely recognized in the academic community, current research is lacking in depth and breadth (Sigwalt et al., 2011). On the one hand, the antidepressant effect of physical exercise may not be significant, as evidenced by its inability to significantly predict depression incidence in adolescents or reduce depressive symptoms in adults, potentially due to heterogeneity in the research results stemming from selection bias (Toseeb et al., 2014; Camacho et al., 1991; Larun et al., 2006). On the other hand, the antidepressant effect of physical exercise serves as a complementary tool in mainstream psychological health promotion and clinical treatment due to the intricate nature of the dose–response relationship (Correia et al., 2024). This is because there is insufficient understanding of how physical exercise impacts depression among college students. Therefore, enhancing research on the antidepressant mechanisms of physical exercise is crucial for improving its antidepressant efficacy among college students in the future.

In recent years, there has been growing interest in understanding the antidepressant effects of physical exercise, leading many scholars to investigate how physical exercise can predict depression in a negative way. For instance, studies have shown that body self-esteem serves as a mediator through which physical exercise negatively predicts depression levels in college students. Meanwhile, physical exercise can decrease the likelihood of depression in college students by affecting self-concept, social support, or both factors (Xu, 2021). Moreover, the interaction between trait mindfulness and social support mediated the impact on depression rates among college students. This body of research not only outlines the antidepressant pathway of physical exercise but also enhances our understanding of the relationship between physical exercise and mental health, providing a foundation for further exploration of additional pathways linking physical exercise and depression in college students. It is important to note that the aforementioned studies examined how various factors influence the mental health of college students from a cross-sectional perspective, thereby overlooking a thorough investigation of longitudinal data. In this regard, some researchers have sought to utilize longitudinal data to demonstrate that the mental health of college students evolves over time (Liu et al., 2023). Conversely significant heterogeneity in mental health exists among different groups.

The shift in psychological research toward positive emotions has led to a growing recognition of the positive impact of positive psychology on mental health issues such as anxiety and depression. Scholars have been particularly interested in the role of positive psychological capital, including self-efficacy, optimism, hope, and resilience, in reducing the likelihood of depression among college students (Tang, 2012; Ge et al., 2006; Chen and Chen, 2008). Moreover, physical exercise has been found to enhance students' positive psychological capital by boosting their satisfaction, sense of achievement, and enjoyment in sports (Yang et al., 2013; Dong et al., 2023). However, existing research has not thoroughly explored he specific mechanisms by which physical exercise affects college students' psychological traits, including self-esteem and positive psychological capital, in relation to depression. This study aims to address this gap by examining how physical exercise can prevent and alleviate depression among college students. The goal is to inform initiatives that promote mental health, enhance social engagement, and improve the overall wellbeing of college students through the antidepressant effects of physical activity.

## 2 Literature review and research hypotheses

### 2.1 The relationship between physical exercise and depression among college students

Numerous scholars have indicated that engaging in physical exercise can enhance mental health, boost self-confidence, and increase life satisfaction, consequently reducing feelings of anxiety and depression (Wu et al., 2016; Esmaeilzadeh, 2015). According to Kyle's study, aerobic exercise has a greater antidepressant impact than resistance exercise among participants, with the combination of aerobic and resistance exercise proving to be the most beneficial (Miller et al., 2020; Mutrie, 2003; Schuch et al., 2016). Evidently, consistent long-term physical activity plays a crucial role in alleviating symptoms of depression and anxiety while also enhancing an individual's self-esteem and optimistic outlook on life (Sun et al., 2014). In terms of research methodologies, intervention research has shown positive effects on sub-health depression and severe depression, although no significant difference was observed between the antidepressant effects of physical exercise, psychological interventions, and drug treatments (Nyström et al., 2015). Cross-sectional studies have demonstrated that regular physical activity among sedentary individuals not only reduces the risk of depression but also decreases the likelihood of developing other mental disorders (Goodwin, 2003). Longitudinal studies have shown that sustained physical exercise over an extended period can be a strong predictor of depression among inactive elderly individuals. Furthermore, physical exercise can enhance the mental wellbeing of older adults by improving their physical health and increasing their level of social support, which subsequently reduces the incidence of depression (Ku et al., 2009). Regarding the design of exercise programs, engaging in moderate-intensity or high-intensity aerobic exercise for 30 min per session, twice a week, has been shown to significantly decrease the risk of depression (Yang et al., 2023). Additionally, in the gender comparisons have indicated that women are more susceptible to depression (Legrand and Heuze, 2007). However, regular physical exercise can significantly reduce the frequency of anxiety and depression in both men and women. In conclusion, physical exercise can directly decrease the incidence of anxiety and depression among college students (Khanzada et al., 2015). The following hypothesis is proposed: H1—Physical exercise has a negative predictive effect on depression among college students.

### 2.2 Relationships among physical exercise, self-esteem, and depression among college students

Self-esteem encompasses the positive self-emotional experience derived from social comparison, comprising self-efficacy, self-worth, self-acceptance, and self-love (Zhang, 2002; Doré, 2017). Scholars have suggested that physical exercise acts as a partial mediator by improving self-esteem levels, thereby ameliorating negative emotions such as depression (Landaas, 2005). Notably, the impact of physical exercise is more pronounced in women, leading to reduced anxiety and depression by bolstering their self-esteem. At the micro level, physical exercise fosters the development of self-efficacy, boosting overall self-esteem through heightened perceived endurance and consequently alleviating depressive symptoms in adult individuals, including elderly individuals. For instance, a 6-month walking program enhanced the self-esteem of elderly individuals, thereby reducing their depression (McAuley et al., 2000). Similarly, physically active interventions have been shown to be beneficial for clinically depressed women, elevating their self-esteem and alleviating depressive symptoms. In summary, physical exercise has been shown to reduce depression in college students directly and by boosting self-esteem (Clark, 2002). However, the generalizability of these findings to broader populations warrants further investigation. Hypothesis H2 is proposed: Self-esteem plays a partial mediating role in the relationship between physical exercise and depression among college students.

### 2.3 Relationships among physical exercise, positive psychological capital, and depression among college students

Positive psychological capital is a stable psychological trait that individuals gradually develop through their personal growth experiences. It consists of four dimensions: self-efficacy, optimism, resilience, and hope (Luthans and Youssef, 2004; Luthans et al., 2015). This trait has been shown to have a positive impact on individuals' work performance, attitudes, and mental health (Luthans et al., 2005; Larson and Luthans, 2006; Alkire and Avey, 2013).

First, positive psychological capital has been shown to improve the mental health of various research groups (Elliott and Fry, 2021). For instance, it studied depressed individuals and found that enhancing positive psychological capital can boost individuals' positive attitudes toward stress, instilling a sense of hope and significantly alleviating negative emotions (Song, 2014). Similarly, it focused on white-collar workers and discovered that higher levels of psychological capital can decrease feelings of loneliness in their work life (Yang, 2008). It conducted research on nurses and reported that increasing nurses' positive psychological capital can lower the likelihood of experiencing psychological issues such as anxiety and depression, especially in high-intensity and high-pressure work environments (Sun and Yang, 2015; Qin and Yuan, 2019). Additionally, Studies have shown that physical exercise can elevate the level of positive psychological capital among college students. This is achieved through the enhancement of positive emotions and resilience, providing students with a competitive advantage in psychological capital and ultimately boosting their overall positive psychological capital. Furthermore, physical exercise can contribute to improved body control, self-efficacy, and optimism among college students. Both moderate- and high-intensity exercise have been found to increase the level of psychological capital in students, with the degree of improvement in positive psychological capital correlating with the amount of exercise undertaken. Finally, it has been suggested that psychological capital may act as a mediator in the relationship between physical exercise and depressive symptoms. Physical exercise can reduce the occurrence of psychological issues such as anxiety and depression by enhancing positive psychological capital. This suggests that positive psychological capital could mediate the relationship between physical exercise and depression in college students (Hegberg and Tone, 2015). However, further investigation is needed to determine whether this mediating role persists in the presence of multiple influencing factors. Hypothesis H3 is proposed: Positive psychological capital plays a partial mediating role in the relationship between physical exercise and depression among college students.

### 2.4 Relationships among physical exercise, self-esteem, positive psychological capital, and depression among college students

The analysis revealed that self-esteem and positive psychological capital each act as separate mediators between physical exercise and depression in college students. Previous studies have indicated a strong correlation between self-esteem and positive psychological capital, suggesting a potential chain mediating role in the relationship between physical exercise and depression among college students (Zhang et al., 2010). However, no existing research has explored this specific relationship. Current studies have established two complementary research paths regarding the influence of self-esteem and positive psychological capital on each other. First, positive psychological capital has a positive impact on self-esteem (Zhong, 2020). For instance, it conducted a study using prisoners as research subjects and found that positive psychological capital reduces aggression in prisoners by mediating self-esteem and impulsivity, as well as through the chain mediating effect of both factors. Similarly, Studies have shown that enhancing positive psychological capital in 3rd-year students can lead to improvements in their self-esteem and mental health (Zhang et al., 2016). Additionally, self-esteem has a positive influence on positive psychological capital. Self-esteem, which involves an individual's positive assessment and perception of self-worth and abilities, contributes to positive psychological capital by fostering a positive self-evaluation that encourages positive attributions, optimism, and realism (Hao and Cui, 2007). This, in turn, motivates individuals to overcome challenges, effectively manage negative life events, foster harmonious relationships with their environment, and expedite social reintegration (Xu and Kuang, 2017). And further research demonstrated that junior high school students with high self-esteem exhibit higher levels of positive psychological capital (Wang, 2019). In essence, higher self-evaluations and the fulfillment of self-needs lead to a stronger sense of accomplishment and increased self-esteem, consequently elevating an individual's positive psychological capital. And individuals with high self-esteem exhibit characteristics of objective understanding and self-acceptance, resulting in a corresponding increase in positive psychological capital (Xi, 2021; Yan, 2022). The study utilized middle school students as research participants and discovered that family functioning can have a positive impact on the prosocial behavior of middle school students. This influence is achieved through the independent mediating role of self-esteem and psychological capital, as well as the sequential mediating effect of both factors. In summary, there is a correlation between self-esteem and positive psychological capital, suggesting a potential sequential connection where self-esteem influences positive psychological capital. Consequently, there might be a mediating chain effect of self-esteem and positive psychological capital on the relationship between physical exercise and depression among college students. Hypothesis H4 is proposed: self-esteem and positive psychological capital act as chain mediators in the correlation between physical exercise and depression among college students.

**Figure 1:**
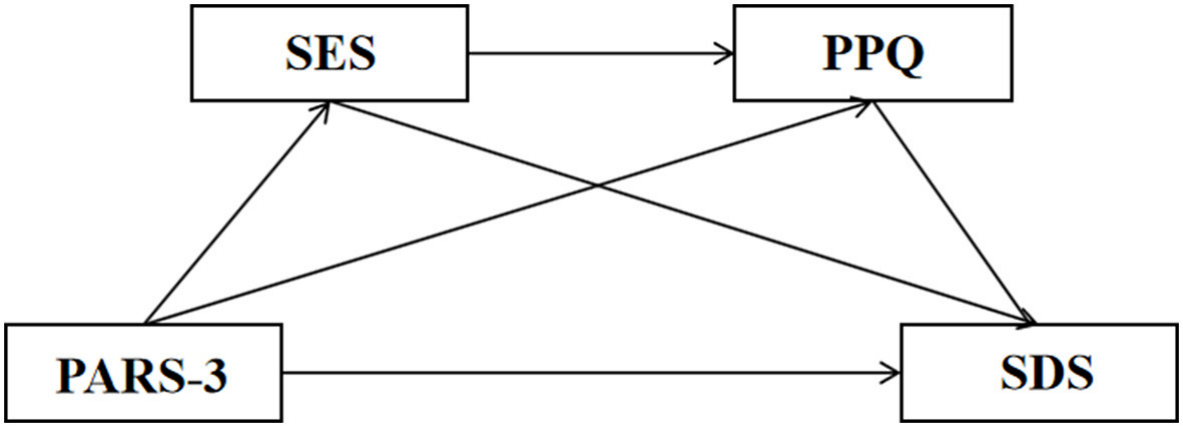
Hypothetical model of physical exercise, self-esteem, positive psychological capital, and depression.

Based on research hypotheses H1–H4, a chain mediation model is proposed to examine the influence of physical exercise on college students' depression through the mediating roles of self-esteem and positive psychological capital, as illustrated in Figure 1.

## 3 Research design

### 3.1 Survey objects and procedures

This article focuses on investigating the impact of physical exercise on depression among college students while considering the mediating roles of self-esteem and positive psychological capital. The study included 1 independent variable (physical exercise), 2 mediating variables (self-esteem and positive psychological capital), and 1 outcome variable (depression). The sample size calculation was based on G-power 3.1.9.7 multiple regression. With reference to previous research findings and specific variable settings (tails = one, H1ρ2 = 0.7, H0ρ2 = 0.5, effect size f2 = 0.15, α = 0.05, power = 0.095, number of predictors = 3), the calculated sample size was 105. To account for potential subject loss during questionnaire completion, the number of distributed questionnaires was increased by 20%, resulting in a minimum sample size of 126. The study targeted freshmen, sophomores, and juniors at two universities in Guangdong. As shown in Table 1, out of 800 distributed questionnaires, 696 were recovered, yielding a recovery rate of 72.38%. Among the 579 valid questionnaires (with an effective rate of 83.19%), there were 132 male and 447 female respondents−480 freshmen and 99 sophomores/juniors—and 216 from urban and 363 from rural areas. Exclusion methods for invalid questionnaires include: (1) uniform responses across the entire questionnaire; (2) failure to select one or more items; and (3) excessive similarity in the responses of two or more questionnaires.

**Table 1:** Distribution of demographic variables of the study subjects.

**Variables**	**Categories**	**Number of people**	**Percentage (%)**
Gender	Male	132	22.80
	Female	447	77.20
Grade	Freshmen	480	82.90
	Sophomores and juniors	99	17.10
Source of students	Urban areas	216	37.31
	Rural areas	363	62.69
Total		579	100

### 3.2 Research tools

#### 3.2.1 Physical Activity Rating Scale-3

The Behavioral Regulation in Exercise Questionnaire (BREQ-3) has been validated in various contexts, demonstrating strong psychometric properties (Cavicchiolo et al., 2022), and could be considered for future research to assess behavioral motivations related to physical activity in university students. The physical activity level of the college students was assessed using Liang Deqing's revised Physical Activity Rating Scale-3 (PARS-3; Liang, 1994). This scale comprises three dimensions—exercise intensity, exercise time, and exercise frequency—each represented by a single question. According to the Likert 5-point scoring system, exercise intensity and frequency are rated from 1 to 5 points, while exercise time is rated from 0 to 4 points. The calculation method for determining college students' physical exercise volume involves multiplying exercise intensity by exercise frequency and then by exercise time, resulting in a score ranging from 0 to 100. A score of 0–19 points indicates a small exercise volume, 20–42 points a medium exercise volume, and 43–100 points a large exercise volume. The Cronbach's alpha coefficient for the physical activity scale used in this study among college students was calculated to be 0.862, indicating a high level of reliability.

#### 3.2.2 Self-Rating Depression Scale

The depression level of college students were assessed using the Self-Rating Depression Scale (SDS), originally developed by William K. Zung and revised by Zhang Mingyuan. This scale encompasses four dimensions: psychotic-affective symptoms, somatic disorders, psychomotor disorders, and depressed psychology (Yuanyuan et al., 2011). It consists of 20 questions, with 10 items scored in a forward manner and 10 in a reverse manner, using a scale of 1–4. To determine the severity of depression, college students sum the positive scores of each item, which are then multiplied by 1.25 to obtain a standard score ranging from 25 to 100 points. Scores between 0 and 50 are considered normal, scores from 50 to 59 indicate mild depression, scores from 60 to 69 indicate moderate depression, and scores above 70 indicate severe depression. In this study, the Cronbach's α coefficient for the Self-Rating Depression Scale among college students was found to be 0.796, demonstrating the scale's high reliability.

#### 3.2.3 Self-Esteem Scale

The self-esteem levels of college students were assessed using the Self-Esteem Scale (SES) developed by Rosenberg and later revised by Li Yifu and Yu Xin (Yang and Wang, 2007). This scale consists of 10 questions measuring two dimensions, self-affirmation and self-denial, with scores ranging from 1 to 4. The total score ranges from 10 to 40, with higher scores indicating higher self-esteem among college students. The Cronbach's α coefficient for the self-esteem scale in this study was 0.887, suggesting a high level of reliability.

#### 3.2.4 Positive Psychological Capital Questionnaire

The level of positive psychological capital among college students was assessed using the Positive Psychological Capital Questionnaire (PPQ) scale developed by Zhang et al. (2010), which consists of four dimensions: optimism, resilience, self-efficacy, and hope. The scale comprises a total of 26 questions rated on a 7-point Likert scale ranging from “completely inconsistent” to “completely consistent,” with scores ranging from 1 to 7. Higher scores indicate a greater level of positive psychological capital among college students. The Cronbach's α coefficient for the Positive Psychological Capital Questionnaire (PPQ) in this study was 0.864, demonstrating high reliability.

### 3.3 Mathematical processing

Excel 2019 was utilized for data entry and reverse scoring, followed by SPSS 26.0 for descriptive analysis, correlation analysis, regression analysis, and chain mediation analysis. The mediation effect analysis was carried out using the bootstrap method of the SPSS macro program Process (version 4.1) plug-in, with a specific indirect effect test model, No. 6, set as X = physical exercise, M1 = self-esteem, M2 = positive psychological capital, and Y = depression; bootstrap samples were set to 5,000 times. Demographic variables of the subject group were controlled for in the stepwise regression analysis to maintain study fairness, ensuring that they were not the main focus of discussion in this article.

## 4 Results

### 4.1 Common method deviation test

In this study, prior to the official distribution of questionnaires, distribution personnel underwent training and guidance to standardize instructions and strictly controlled the duration of the questionnaires and demographic variables to mitigate common method bias (Zhang and Huang, 2017). However, the influence of factors such as the environment and guidance context during scale completion remained uncontrollable (Hu and Gan, 2008). Consequently, Harman's single-factor test was conducted on the survey data, with unrotated principal component analysis performed using SPSS 26.0 on physical exercise, self-esteem, positive psychological capital, and depression. The results revealed 12 factors with characteristic roots >1, with the first factor explaining 8.28% of the variance, falling below the critical value of 40%. This suggests that there is no common method bias present in the measurement data of this study.

### 4.2 Correlations between physical exercise, self-esteem, positive psychological capital, and depressive symptoms

Through descriptive analysis and Pearson correlation analysis of data concerning college students' physical exercise, self-esteem, positive psychological capital, and depression data, the results revealed a significant positive correlation between physical exercise and self-esteem as well as between physical exercise and psychological capital (Table 2). Furthermore, there was a significants were found between physical exercise and depression, between self-esteem and depression, and between psychological capital and depression. These noteworthy correlations among the variables establish a robust foundation for testing the mediating effect.

**Table 2:** Correlation analysis results of physical activity, self-esteem, positive psychological capital, and depression (*n* = 579).

**Variable**	**M ±SD**	**1. PARS-3**	**2. SES**	**3. PPQ**	**4. SDS**
1. PARS-3	8.157 ± 5.943	1			
2. SES	25.599 ± 3.841	0.111^**^	1		
3. PPQ	112.460 ± 19.431	0.281^**^	0.302^**^	1	
4. SDS	42.308 ± 19.431	0.181^**^	0.220^**^	0.272^**^	1

**p < 0.01, the correlation coefficient was significant.

### 4.3 Mediating effect analysis

Using college students' physical exercise as the independent variable, depression as the dependent variable, and self-esteem and positive psychological capital as mediating variables, this study employed the SPSS26.0 and PROCESS4.1 plug-ins to analyze the relationships between these factors while controlling for demographic variables such as gender, age, and place of origin. The chain mediating effect of positive psychological capital on the relationship between physical exercise and depression was examined, and the results are presented in Table 3. The analysis involved several steps. First, a regression model was established with depression as the dependent variable and physical exercise as the independent variable, revealing a significant negative impact of physical exercise on depressive mood (β = −0.181, *P* < 0.001), supporting research hypothesis H1. In the subsequent steps, self-esteem and positive psychological capital were included in the model to assess their roles as mediating variables. The findings indicated that physical exercise, self-esteem, and positive psychological capital all significantly and negatively predicted depression. Moreover, physical exercise positively predicted self-esteem and positive psychological capital, while self-esteem also positively predicted positive psychological capital. The regression coefficients indicate that self-esteem and positive psychological capital play a significant role as partial mediators and chain mediators between physical exercise and depression, supporting H2 and H3. The mediation effects were tested using AMOS 28.0, which demonstrated a good model fit with χ^2^/df = 2.768, CFI = 0.924, TLI = 0.937, RMSEA = 0.065, and SRMR = 0.041.

**Table 3:** Mediating effect regression model of self-esteem and positive psychological capital.

	**SDS**		**SES**		**PPQ**		**SDS**	
	β	***t***	β	***t***	β	***T***	β	***t***
PARS-3	0.181	4.463^***^	0.113	2.725^**^	0.251	6.584^***^	0.109	2.682^**^
SES					0.274	7.183^***^	0.149	3.645^***^
PPQ							0.196	4.644^***^
R	0.181		0.111		0.391		0.325	
*R* ^2^	0.033		0.012		0.153		0.106	
*F*	19.978^***^		7.425^***^		53.397^***^		23.286^***^	

**p < 0.05, the regression coefficient is significant.

***p < 0.001, the regression coefficient is significant.

The bootstrap test was repeated 5,000 times to examine the mediating effects and confidence intervals of self-esteem and positive psychological capital on the relationship between physical exercise and depression (refer to Table 4). The findings revealed that the total effect of physical exercise on depression was 0.205. The 95% confidence intervals for the mediating effects of self-esteem and positive psychological capital (LLCI = −0.295, ULCI = −0.115) are not 0, suggesting that physical exercise has a significant impact on depression. Both the total effect and the mediating effects of self-esteem and positive psychological capital are statistically significant. The direct effect of physical exercise on depression was 0.133, with a bootstrap 95% confidence interval excluding 0 (LLCI = −0.214, ULCI = −0.033), indicating a substantial direct effect of physical exercise on depression, which accounted for 64.90% of the total effect.

**Table 4:** Mediating effects of self-esteem and positive psychological capital.

		**Effect**	**Boot SE**	**Boot LLCI**	**Boot ULCI**	**Ratio of indirect to total effect**
Total effect		0.205	0.045	0.295	0.115	
Direct effect		0.133	0.046	0.214	0.033	0.649
	Total indirect effect	0.072	0.023	0.128	0.033	0.351
Indirect effect	Indirect effect 1 (M1-Y)	0.017	0.012	0.051	0.004	0.083
	Indirect effect 2 (M2-Y)	0.049	0.019	0.088	0.011	0.239
	Indirect effect 3 (M1-M2-Y)	0.006	0.003	0.011	0.001	0.029
	Compare 1	0.037	0.045	0.058	0.151	
	Compare 2	0.012	0.030	0.107	0.001	
	Compare 3	0.049	0.047	0.188	0.013	

**Figure 2:**
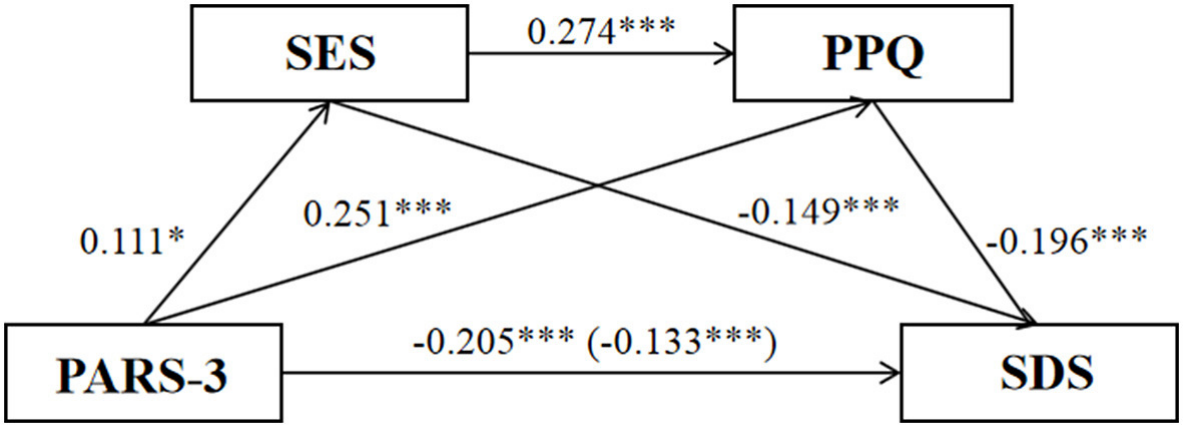
Chain mediation effect model of self-esteem and positive psychological capital. ***p < 0.001, the path coefficient was significant.

This study revealed that physical exercise can be a predictor of depression among college students, with self-esteem and positive psychological capital acting as indirect mediators through three distinct paths, as shown in Figure 2. The total indirect effect is calculated to be 0.072, and the bootstrap 95% confidence interval, ranging from LLCI = −0.128 to ULCI = −0.033, does not encompass 0, representing 35.10% of the total effect. The first mediating path, physical exercise to self-esteem to depression (path 1), exhibited an indirect effect of 0.017, with the bootstrap 95% confidence interval excluding 0 (LLCI = −0.051, ULCI = −0.004) and accounting for 8.30% of the total effect. The second path, physical exercise to positive psychological capital to depression (path 2), had an indirect effect of 0.049, with the bootstrap 95% confidence interval also not containing 0 (LLCI = −0.088, ULCI = −0.011) and contributing to 23.90% of the total effect. The third path, a chain mediation through physical exercise to self-esteem to positive psychological capital to depression (path 3), demonstrated an indirect effect of 0.006, with the bootstrap 95% confidence interval excluding 0 (LLCI = −0.011, ULCI = −0.001) and representing 2.90% of the total effect. These findings support research hypothesis H4. Consequently, a chain mediation model involving self-esteem and positive psychological capital between physical exercise and depression in college students is proposed.

## 5 Discussion

### 5.1 The direct effect of physical exercise on depression among college students

The research findings indicate a correlation between physical exercise and depression among college students, revealing that physical exercise has a significant negative impact on depression levels. These results align with those of previous studies, thereby reinforcing the established relationship between physical activity and depression in this demographic (Heissel et al., 2023; Philippot et al., 2022). The reasons why physical exercise can reduce depression among college students are as follows. Physical exercise can lead to physiological changes in college students, resulting in structural changes in brain function. This is evident through increased cerebral blood flow and the production of physiological hormones from accelerated metabolism, which can help inhibit negative emotions (Mei, 2001). Additionally, physical exercise can suppress negative emotions, improve positive feelings, and reduce depressive symptoms by weakening the response of the HPA axis to stress (Erickson et al., 2012; Cui, 2005). The combination of these effects can offer psychological benefits to college students. Furthermore, physical exercise can effectively divert attention from academic and employment pressures, relax tension, activate individual goals, enhance volitional behavior, and alleviate emotional disorders, ultimately helping to alleviate depressive symptoms (Nolen-Hoeksema, 1991). Finally, leveraging the socioemotional function of physical exercise can facilitate idea exchange, emotional interaction, and interpersonal communication among college students. This can enhance the structure of active social networks, provide social support, and improve emotional regulation, the ability to navigate complex environments, and interpersonal skills, thus reducing depression and mental stress resulting from conflicts among college students (Zhang and Mao, 2003). This perspective is further supported by the social interaction hypothesis, which suggests that college students benefit from sharing information and knowledge with peers during physical exercise. This exchange helps students manage negative emotions, enhances their sense of group belonging, and boosts individual satisfaction. To fully leverage the antidepressant benefits of physical exercise, college students should cultivate regular exercise routines, seek support from exercise companions, and be attuned to the emotional dynamics of their peers during physical activity.

### 5.2 The mediating effect of self-esteem between physical exercise and depression among college students

The findings of this study demonstrate that self-esteem acts as a partial mediator in the connection between physical exercise and depression in college students, with a relative mediation effect of 8.3%. This suggests that physical exercise not only directly decreases depression in college students but also indirectly alleviates it through the enhancement of self-esteem, thus supporting research hypothesis H2. These results align with previous studies indicating that physical exercise boosts overall self-esteem by enhancing body self-esteem, reducing negative self-worth components, and ultimately mitigating depression (Ryan, 2008; Ekeland et al., 2004; Kim and Ahn, 2021). This finding is consistent with the findings of study and other researchers, who highlighted how physical exercise can elevate college students' self-esteem and reduce their levels of depression (Jiang, 2009). Longitudinal data indicate that high self-esteem in college students can serve as a negative predictor for low anxiety and other adverse psychological issues (Cao and Liu, 2024). Additionally, physical exercise boosts students' exercise self-efficacy, enhancing their physical fitness and skills, thereby elevating their sense of self-worth and transforming them into individuals with high self-esteem. College students with high self-esteem tend to be confident, rational, and adaptable to new environments, possessing a positive self-image and effectively filtering out negative external evaluations to mitigate negative social behaviors resulting from academic and career pressures. By reinforcing personal value and meaning, physical exercise can safeguard against mental health issues such as anxiety and depression (Wang et al., 2020). In summary, physical exercise can alleviate depression in college students by bolstering self-esteem.

### 5.3 The mediating effect of positive psychological capital between physical exercise and depression among college students

The results of this study demonstrated a significant correlation between physical exercise, positive psychological capital, and depression in college students. Further investigation revealed that physical exercise can directly reduce the incidence of depression in college students and indirectly through the partial mediating effect of positive psychological capital. These findings support Hypothesis 3, indicating that positive psychological capital serves as a partial mediator between physical exercise and depression among college students, with a relative mediation effect of 28.9%. There is a positive correlation between physical exercise and positive psychological capital and a negative correlation between positive psychological capital and depression, which is consistent with previous research findings (Zhang, 2021). Previous studies have indicated that physical exercise can enhance the positive psychological capital of college students, which is a crucial psychological resource for managing stressful life events and negative feedback (Li et al., 2020). This has significant implications for preventing and addressing negative psychological issues such as depression among college students. Additionally, physical exercise can help change the negative self-perception of college students with depression and boost their self-efficacy by fostering positive peer relationships and social structures. And it can help slow down and mitigate the depletion of psychological energy in college students (Cohen, 2004). The mediating effect mechanism of positive psychological capital in relation to physical exercise is primarily evident in four key aspects. First, engaging in physical exercise helps college students respond to external negative evaluations and boost their self-confidence by enhancing their self-efficacy within positive psychological capital. Second, physical exercise can enhance resilience within positive psychological capital, enabling students to better navigate challenging situations and improve their psychological fortitude (Stanley and Mettilda Bhuvaneswari, 2016). Third, physical exercise addresses college students' uncertainties and fears about future employment by fostering hope within positive psychological capital, thereby enhancing their outlook on future interpersonal interactions and career prospects (Peh et al., 2017). Finally, physical exercise enhances college students' social adaptability by promoting optimistic traits within their psychological capital, aiding in coping with social anxiety stemming from new environments (Dolcos et al., 2016). Consequently, positive psychological capital serves as a mediating factor between physical exercise and depression in college students.

### 5.4 The chain mediating effect of self-esteem and positive psychological capital between physical exercise and depression among college students

This study investigated the influence of physical exercise, self-esteem, and positive psychological capital on depression among college students. The findings revealed a total effect value of β = 0.205. Specifically, physical exercise, self-esteem, and positive psychological capital were all significantly correlated with depressive symptoms. The direct predictive effect on college students' depressive mood was β = 0.133, indicating a total indirect effect value of β = 0.072. This suggests the presence of both partial and chain mediation effects of self-esteem and positive psychological capital, with the third chain mediation path contributing 2.9% to the total effect. The study demonstrated that physical exercise not only independently and negatively predicted depression among college students through self-esteem and positive psychological capital but also exerted a combined impact on depression through the chain mediation of these factors. These results underscore the importance of self-esteem and positive psychological capital in alleviating depression among college students. College students who engage in physical exercise often experience an increase in self-esteem, which in turn has a positive impact on their psychological capital. Research indicates a strong link between high self-esteem and positive psychological capital among college students, with high self-esteem levels serving as a significant predictor of psychological capital. Physical exercise not only boosts self-esteem but also contributes to overall psychological wellbeing. Over time, students tend to view themselves more positively, leading to increased self-confidence and a more optimistic outlook on the future. This improved self-perception helps students face challenges with a positive attitude, ultimately enhancing their psychological capital and alleviating feelings of depression. By promoting self-acceptance, self-challenge, and self-confidence, physical exercise enables college students to perform high-intensity tasks with confidence and resilience, thereby reducing the likelihood of experiencing negative emotions such as depression. In addition to physical exercise, educational interventions such as media literacy programs, have been shown to significantly influence students' psychological wellbeing, particularly within the context of sports science (Mallia et al., 2020). These interventions enables students to appreciate diverse concepts and ideas during emotional interactions, which leads to improved scientific rationality in self-evaluation, enhanced self-esteem, and increased positive psychological capital. Ultimately, this contributes to a reduction in depression among college students. The proposed chain mediation in this study integrates research on the relationships among self-esteem, positive psychological capital, and college students' depression, thereby providing a more comprehensive understanding of how physical exercise impacts depression in this demographic. These findings have implications for advancing reforms in physical education curricula at colleges and universities, offering valuable guidance.

## 6 Conclusions, proposal, and prospects

### 6.1 Conclusion

There was a notable correlation between physical exercise, self-esteem, positive psychological capital, and depression among college students. Physical exercise has a significant negative impact on predicting depressive moods in college students and serves as a crucial variable in influencing their overall mood. Additionally, self-esteem and positive psychological capital not only partially mediate the relationship between physical exercise and depressive moods in college students but also have a cascading effect on their mood through these factors. This indirect mediating effect has a substantial influence on depression among college students.

### 6.2 Proposal

The conclusion of this study indicates that physical exercise is both feasible and universally effective in reducing depression among college students. Consequently, it is essential for the state, society, families, and educational institutions to collaborate in actively promoting physical exercise among Chinese college students. This can be achieved by enhancing their self-esteem through encouragement and other supportive measures during physical activities, thereby striving to alleviate their anxiety related to academic pressures and uncertainties in job searching, ultimately improving their positive psychological capital. Furthermore, in light of the increasing integration of artificial intelligence across various industries, it is beneficial to incorporate advanced information technologies, such as big data analysis and cloud computing, into the physical exercise routines of college students. By employing personalized digital services to monitor and address the emergence of negative emotions, such as depression, the accuracy and effectiveness of mental health services for college students can be significantly enhanced.

### 6.3 Prospects

The impact of physical exercise on depression among college students is significant, with self-esteem and positive psychological capital serving as crucial mediators. Furthermore, these variables exhibit a chain mediating effect. The relationships among these factors appear to revolve around social support, indicating that future research should integrate social support variables to enhance the understanding of the connection between physical exercise and depression in college students. Practically, it is essential to emphasize the role of social sports organizations and clubs, as well as to incorporate public sports courses as platforms for fostering social support among college students participating in physical exercise. This strategy can assist in guiding college students toward developing a robust social network, ultimately contributing to improved self-esteem and positive psychological capital while reducing the incidence of depression.

## 7 Limitations of the study

Geographically limited sample: As the study was conducted in only two universities, the findings may not be generalizable to the broader population of Chinese university students. Self-reports: The reliance on self-administered questionnaires may introduce bias associated with self-perception. Lack of longitudinal data: The study is based on cross-sectional data, which restricts the ability to establish causal relationships.

## Data Availability

The datasets presented in this study can be found in online repositories. The names of the repository/repositories and accession number(s) can be found in the article/supplementary material.
